# Polyethylenimine-Coated Ultrasmall Holmium Oxide Nanoparticles: Synthesis, Characterization, Cytotoxicities, and Water Proton Spin Relaxivities

**DOI:** 10.3390/nano12091588

**Published:** 2022-05-07

**Authors:** Shuwen Liu, Huan Yue, Son Long Ho, Soyeon Kim, Ji Ae Park, Tirusew Tegafaw, Mohammad Yaseen Ahmad, Seungho Kim, Abdullah Khamis Ali Al Saidi, Dejun Zhao, Ying Liu, Sung-Wook Nam, Kwon Seok Chae, Yongmin Chang, Gang Ho Lee

**Affiliations:** 1 Department of Chemistry, College of Natural Sciences, Kyungpook National University, Taegu 41566, Korea; liushuwen0701@gmail.com (S.L.); yuehuan888@gmail.com (H.Y.); sonlongh@gmail.com (S.L.H.); tirukorea@gmail.com (T.T.); yaseen.knu@gmail.com (M.Y.A.); abdullah_al_saidi@hotmail.com (A.K.A.A.S.); djzhao.chem@gmail.com (D.Z.); ly1124161@gmail.com (Y.L.); 2 Division of RI-Convergence Research, Korea Institute of Radiological and Medical Sciences (KIRAMS), Seoul 01817, Korea; ksy0188@kirams.re.kr (S.K.); jpark@kirams.re.kr (J.A.P.); 3 Department of Medical & Biological Engineering, Kyungpook National University, Taegu 41944, Korea; seungho5335@gmail.com; 4 Department of Molecular Medicine, School of Medicine, Kyungpook National University, Taegu 41944, Korea; nams@knu.ac.kr; 5 Department of Biology Education, Teachers’ College, Kyungpook National University, Taegu 41566, Korea; kschae@knu.ac.kr

**Keywords:** Ho_2_O_3_, ultrasmall nanoparticle, polyethylenimine coating, relaxivity, cytotoxicity

## Abstract

Water proton spin relaxivities, colloidal stability, and biocompatibility of nanoparticle magnetic resonance imaging (MRI) contrast agents depend on surface-coating ligands. In this study, hydrophilic and biocompatible polyethylenimines (PEIs) of different sizes (M_n_ = 1200 and 60,000 amu) were used as surface-coating ligands for ultrasmall holmium oxide (Ho_2_O_3_) nanoparticles. The synthesized PEI1200- and PEI60000-coated ultrasmall Ho_2_O_3_ nanoparticles, with an average particle diameter of 2.05 and 1.90 nm, respectively, demonstrated low cellular cytotoxicities, good colloidal stability, and appreciable transverse water proton spin relaxivities (r_2_) of 13.1 and 9.9 s^−1^mM^−1^, respectively, in a 3.0 T MR field with negligible longitudinal water proton spin relaxivities (r_1_) (i.e., 0.1 s^−1^mM^−1^) for both samples. Consequently, for both samples, the dose-dependent contrast changes in the longitudinal (R_1_) and transverse (R_2_) relaxation rate map images were negligible and appreciable, respectively, indicating their potential as efficient transverse T_2_ MRI contrast agents in vitro.

## 1. Introduction

Nanoparticles are ideal materials for use as negative magnetic resonance imaging (MRI) contrast agents because they have sufficient magnetic moments at room temperature to generate considerable local magnetic field fluctuations to induce transverse (T_2_) water proton spin relaxations [1,2,3,4]. Consequently, they can provide negative (or darker) contrasts in MR images. However, molecular agents containing one metal ion at the chelating coordination center have paramagnetic moments that are extremely small. Their small paramagnetic moments do not induce sufficient T_2_ water proton spin relaxations to provide negative contrasts in MR images at typical injection concentrations [4,5].

Certain lanthanide oxide nanoparticles are suitable MRI contrast agents because their paramagnetic moments at room temperature are sufficiently high to induce water proton spin relaxations [6,7,8,9,10,11,12], especially at high MR fields [10,11,13,14]. Their magnetic moments are attributed to 4f-electron spin-orbital or spin motions [15]. The 4f-electrons have compact 4f-orbitals and are shielded by 5s- and 5p-orbitals; hence, these magnetic moments are almost unaffected by surface effects such as coating ligand and particle size [16]. Therefore, magnetic moments (unit: emu/g) of lanthanide oxide nanoparticles are nearly independent of the coating ligand and particle size. Importantly, because they are almost independent from particle size, lanthanide oxide nanoparticles can function as MRI contrast agents even at ultrasmall particle diameters (<3 nm) at which nanoparticles are excretable via the renal system as in the case of molecular agents [17,18].

In this study, ultrasmall holmium oxide (Ho_2_O_3_) nanoparticles were examined because of their unique magnetic properties obtained from Ho^3+^ (^5^I_8_) [15]. Ho^3+^ is a lanthanide ion with high magnetic moment [15]. Thus, ultrasmall Ho_2_O_3_ nanoparticles have an appreciable magnetic moment at room temperature [9,10,11]. Interestingly, because of the contribution of the fast 4f-electron orbital motion to the magnetic moment of Ho^3+^ and the mismatch between the fast 4f-electron motion of Ho^3+^ and slow water proton spin motion, nanoparticles can exclusively cause only T_2_ water proton spin relaxations with negligible longitudinal (T_1_) water proton spin relaxations [19]. Hence, the nanoparticles can only cause negative contrasts in MR images, thus acting as efficient T_2_ MRI contrast agents [2].

In MRI contrast agents, surface-coating ligands play an important role because they affect the water proton spin relaxivities, colloidal stability, and biocompatibility of contrast agents [7,20,21,22,23]. Compared with small molecules, polymers have many hydrophilic groups for binding to nanoparticles; thus, they can show improved colloidal stability, better biocompatibility, and thicker coating for the nanoparticles [20,23]. In this study, polyethylenimine (PEI) was used as a surface-coating ligand because it has many hydrophilic primary amines useful for binding to nanoparticles [24,25,26,27,28,29,30] and demonstrates good biocompatibility [31,32]. PEI has many secondary and tertiary amine groups that enhance the hydrophilicity of the nanoparticles. Therefore, PEI has been extensively used as a surface-coating ligand for various nanoparticles in biomedical applications such as drug delivery [33,34], imaging [35,36,37], cancer cell separation [38], cancer therapy [39,40], and gene delivery [41,42,43,44,45,46].

Herein, PEI-coated ultrasmall Ho_2_O_3_ nanoparticles were synthesized using a one-pot polyol method. In previous studies [9,10,11], D-glucuronic acid- and polyacrylic acid-coated ultrasmall Ho_2_O_3_ nanoparticles had been investigated as efficient T_2_ MRI contrast agents. However, physicochemical properties such as zeta potentials, hydrodynamic diameters, colloidal stability, and cellular cytotoxicities and MRI properties such as water proton spin relaxivities depend on surface-coating ligands. Therefore, PEI polymers were used as surface-coating ligands in this study to explore the physicochemical properties and water proton spin relaxivities of PEI-coated ultrasmall Ho_2_O_3_ nanoparticles. This study will enhance the importance of surface-coating ligands in T_2_ MRI contrast agents. PEI polymers of different sizes, namely, PEI1200 and PEI60000 (M_n_ = 1200 and 60,000 amu, respectively) were used as surface coating ligands. We characterized PEI-coated ultrasmall Ho_2_O_3_ nanoparticles using multiple experimental techniques. Biocompatibility was verified by cellular cytotoxicity measurements. To explore the potential of synthesized nanoparticles as efficient transverse T_2_ MRI contrast agents in vitro, longitudinal (r_1_) and transverse (r_2_) water proton spin relaxivities and longitudinal (R_1_) and transverse (R_2_) relaxation rate map images were obtained in a 3.0 T MR field.

## 2. Materials and Methods

### 2.1. Chemicals

Chemicals, such as Ho(NO_3_)_3_∙5H_2_O (99.9%), NaOH (>99.9%), triethylene glycol (TEG; 99%), PEI [50 wt.% in water, M_n_ = 1200 amu (M_w_ = 1300 amu; PEI1200) and 60,000 amu (M_w_ = 750,000 amu; PEI60000)], dimethyl sulfoxide (DMSO) (99.9%), and Rosewell Park Memorial Institute (RPMI)1640 culture medium were obtained from Sigma-Aldrich (Burlington, MA, USA) and used as-received. Ethanol (99.5%) was purchased from Duksan (Ansan, South Korea) and used as-received for the initial washing of nanoparticles. Triple-distilled water was used for the final washing of nanoparticles and for preparing nanoparticle suspension samples (~30 mM Ho).

### 2.2. One-Pot Polyol Synthesis of PEI-Coated Ultrasmall Ho_2_O_3_ Nanoparticles

**Figure 1:**
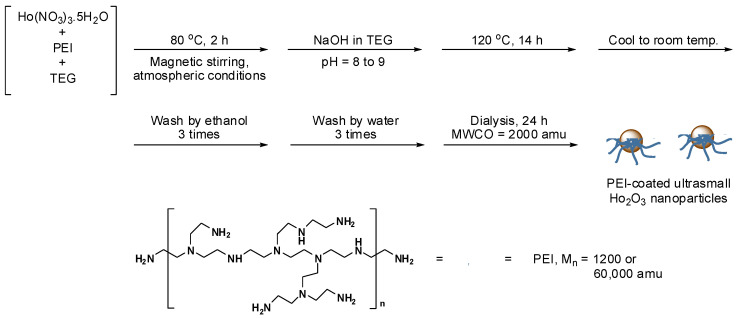
One-pot polyol synthesis of ultrasmall Ho_2_O_3_ nanoparticles coated with PEI1200 and PEI60000 (M_n_ = 1200 and 60,000 amu, respectively).

Figure 1 shows the one-pot polyol synthesis of PEI-coated ultrasmall Ho_2_O_3_ nanoparticles. In brief, 2.0 mmol of Ho(NO_3_)_3_∙5H_2_O and PEI (1.0 mmol of PEI1200 or 0.02 mmol of PEI60000) was dissolved in 20 mL of TEG in a three-necked round bottom flask placed inside a stirring heating mantle (MS-DMS, Misung Scientific Co. Ltd., Seoul, South Korea) at 80 °C for 2 h under normal atmospheric conditions. Separately, 7.0 mmol of NaOH was added to 15 mL of TEG at 80 °C with magnetic stirring until NaOH was completely dissolved in TEG, and the prepared NaOH solution was slowly dropped into the aforementioned precursor solution with magnetic stirring until the pH of the solution reached ~9. After the solution pH became nearly constant, the reaction temperature increased to 120 °C and was maintained at that temperature for 14 h with magnetic stirring. Then, the solution was air-cooled to room temperature. To remove the unreacted precursors, Na^+^, OH^−^, PEI, and TEG from the product nanoparticles, 400 mL of ethanol was added to the product solution, which was then magnetically stirred for 10 min. The solution was then placed in a refrigerator (~4 °C) until the nanoparticles settled at the bottom of the beaker. The top transparent solution was decanted. This washing process with ethanol was repeated thrice. To remove ethanol from the product’s nanoparticles, 400 mL of triple-distilled water was added to the product solution, which was then concentrated to ~20 mL using a rotary evaporator. For additional purification of nanoparticles, the product solution was dialyzed against 1 L of triple-distilled water using a dialysis tube (molecular weight cutoff (MWCO) = ~2000 amu) for 24 h with magnetic stirring.

### 2.3. General Characterizations

#### 2.3.1. Particle Diameters

The particle diameter of the PEI-coated ultrasmall Ho_2_O_3_ nanoparticles was determined by high-resolution transmission electron microscopy (HRTEM) using the Titan G2 ChemiSTEM CS Probe (200 kV; FEI, Hillsboro, OR, USA). For measurements, a drop of the diluted nanoparticle sample in ethanol was dropped on a carbon film supported by a 200-mesh copper grid using a micropipette (2–20 μL, Eppendorf, Hamburg, Germany) and allowed to dry in air at room temperature. The copper grid with nanoparticles was then placed in the vacuum chamber of the microscope for characterization.

#### 2.3.2. Metal Concentrations in the Aqueous Nanoparticle Suspension Samples

The Ho concentration of the nanoparticle suspension sample in the aqueous media was determined by inductively coupled plasma-atomic emission spectroscopy (ICP-AES) using the IRIS/AP spectrometer (Thermo Jarrell Ash Co., Waltham, MA, USA).

#### 2.3.3. Hydrodynamic Diameters

A dynamic light scattering (DLS) particle size analyzer (Zetasizer Nano ZS, Malvern, Malvern, UK) was used to measure the hydrodynamic diameters and zeta potentials of the nanoparticle suspension samples in aqueous media (~0.5 mM Ho).

#### 2.3.4. Nanoparticle Crystal Structures

A multi-purpose X-ray diffractometer (X’PERT PRO MRD, Philips, Amsterdam, The Netherlands) with unfiltered CuKa radiation (λ = 0.154184 nm) was used to characterize the crystal structures of the nanoparticle powder samples. The scanning step and scan range in 2θ were 0.033° and 15–100°, respectively.

#### 2.3.5. Surface-Coating Analyses

The attachment of PEI polymers to the Ho_2_O_3_ nanoparticles was probed by obtaining the Fourier transform infrared (FT-IR) absorption spectra (Galaxy 7020A, Mattson Instrument Inc., Madison, WI, USA) and using the powder samples pelletized with KBr. The scan range was 400–4000 cm^−1^. A thermogravimetric analysis (TGA) instrument (SDT-Q600, TA Instrument, New Castle, DE, USA) was used to estimate the surface-coating wt.% of ligands in the sample by recording the TGA curves between room temperature and 900 °C under air flow. The average amount of surface-coated ligands was estimated from mass loss after considering water and air desorption between room temperature and ~105 °C. Then, the amount of Ho_2_O_3_ nanoparticles in the sample was estimated from the remaining mass. After TGA, each sample was collected and subjected to X-ray diffraction (XRD) analysis.

#### 2.3.6. Magnetic Property Measurements

A vibrating sample magnetometer (7407-S, Lake Shore Cryotronics Inc., Westerville, OH, USA) was used to characterize the magnetic properties of nanoparticle samples by recording magnetization (M) versus applied field (H) (or M−H) curves (−2.0 T ≤ H ≤ 2.0 T) at 300 K. The measurements were performed using powder samples of 20–30 mg; the net M value of each sample (i.e., only the Ho_2_O_3_ nanoparticles without the PEI coating) was estimated using the net mass of Ho_2_O_3_ nanoparticles obtained from the TGA curve.

### 2.4. In Vitro Cellular Cytotoxicity Measurements

The in vitro cellular cytotoxicities of PEI-coated nanoparticles were measured using the CellTiter-Glo Luminescent Cell Viability Assay (Promega, Madison, WI, USA). The intracellular adenosine triphosphate was quantified using a Victor 3 luminometer (Perkin Elmer, Waltham, MA, USA). The human prostate cancer (DU145) cell line (Korean Cell Line Bank, Seoul, Korea) was used. The RPMI1640 was used as a cell culture medium. The cells were seeded on a separate 24-well cell culture plate and incubated for 24 h. Five test sample solutions (10, 50, 100, 200, and 500 μM Ho) were prepared by diluting the concentrated original nanoparticle suspension samples with a sterile phosphate-buffered saline solution and 2 mL aliquots were used to treat the cells, which were subsequently incubated for 48 h. Cell viabilities were measured thrice to obtain the average cell viabilities, which were then normalized in terms of the viability of untreated control cells (0.0 mM Ho).

### 2.5. Water Proton Spin Relaxivity Measurements

T_1_ and T_2_ water proton spin relaxation times and R_1_ and R_2_ map images were measured using a 3.0 T MRI scanner (GE 3.0 T Signa Advantage, GE Medical Systems, Chicago, IL, USA). Aqueous dilute solutions (1, 0.5, 0.25, 0.125, and 0.0625 mM Ho) were prepared by diluting the concentrated aqueous nanoparticle suspension samples (~30 mM Ho) with triple-distilled water. These dilute solutions were used to obtain both T_1_ and T_2_ relaxation times and R_1_ and R_2_ map images. Then, r_1_ and r_2_ water proton spin relaxivities were estimated from the slopes of the plots of the inverse relaxation times 1/T_1_ and 1/T_2_ versus Ho concentration, respectively. T_1_ relaxation time measurements were performed using an inversion recovery method. In this method, the inversion time (TI) was varied at 3.0 T, and the MR images were acquired at 34 TI values in the range of 50−1750 ms. Then, T_1_ relaxation times were obtained from the nonlinear least-square fits to the measured signal intensities at multiple TI values. The parameters used in T_1_ relaxation time measurements were as follows: slice thickness = 8 mm, repetition time (TR) = 2000 ms, echo time (TE) = 28 ms, echo train length (ETL) = 17, flip angle = 120°, matrix size = 320 × 256, and field of view (FOV) = 250 × 200 mm. For T_2_ relaxation time measurements, the Carr–Purcell–Meiboom–Gill pulse sequence was used for multiple spin-echo measurements. Then, 32 images were acquired at 32 TE values in the range of 15–480 ms. T_2_ relaxation times were obtained from the nonlinear least-square fits to the mean pixel values for the multiple spin-echo measurements at multiple TE values. The parameters used in T_2_ relaxation time measurements were as follows: slice thickness = 8 mm, TR = 2000 ms, ETL = 1, flip angle = 180°, matrix size = 320 × 256, and FOV = 250 × 200 mm.

## 3. Results

### 3.1. Physicochemical Properties of the PEI-Coated Ultrasmall Ho_2_O_3_ Nanoparticles

**Figure 2:**
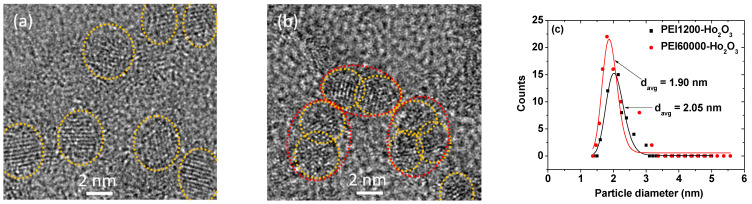

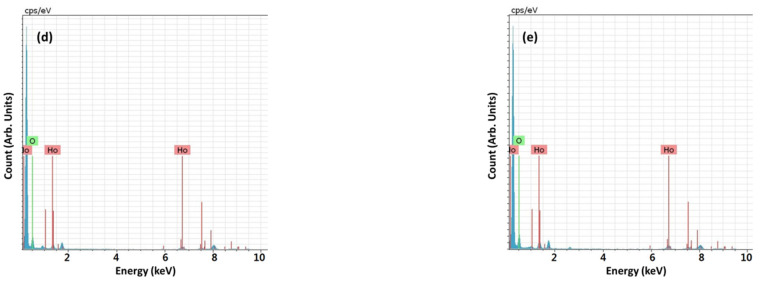
HRTEM images of (**a**) PEI1200- and (**b**) PEI60000-coated ultrasmall Ho_2_O_3_ nanoparticles. Dotted circles indicate individual nanoparticles. Large dotted circles in (**b**) indicate nanoparticles grafted together with one PEI60000. (**c**) Particle diameter distributions and log-normal function fits of PEI1200- and PEI60000-coated ultrasmall Ho_2_O_3_ nanoparticles. EDS spectra of (**d**) PEI1200- and (**e**) PEI60000-coated ultrasmall Ho_2_O_3_ nanoparticles.

PEI1200- and PEI60000-coated ultrasmall Ho_2_O_3_ nanoparticles were synthesized using a polyol method [47] and characterized using various experimental techniques. As shown in the HRTEM images (Figure 2a,b), the particle diameters of the synthesized nanoparticles (labeled with dotted circles) were ultrasmall and ranged from 1.0 to 3.5 nm. For PEI60000-coated nanoparticles, approximately two nanoparticles appeared to be grafted with one PEI60000 because of the large size of PEI60000 (labeled with large dotted circles in Figure 2b), as discussed further in Section 3.2. The average particle diameter (d_avg_) of the PEI1200- and PEI60000-coated Ho_2_O_3_ nanoparticles was estimated to be 2.05 and 1.90 nm, respectively, from the log-normal function fits to the observed particle diameter distributions (Figure 2c and Table 1). Furthermore, the results of energy-dispersive X-ray spectroscopy (EDS) substantiated the presence of C, O, and Ho in PEI1200- and PEI60000-coated Ho_2_O_3_ nanoparticles (Figure 2d,e).

**Figure 3:**
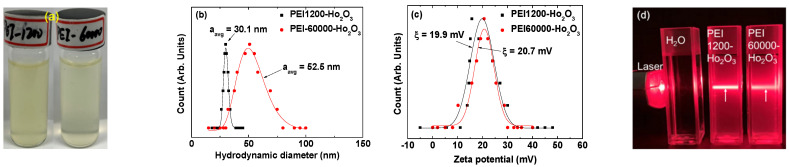
(**a**) Images of PEI1200- and PEI60000-coated ultrasmall Ho_2_O_3_ nanoparticles in aqueous media (vials on the left and right-side, respectively) with a concentration of ~30 mM Ho. (**b**) DLS patterns of PEI1200- and PEI60000-coated ultrasmall Ho_2_O_3_ nanoparticles in aqueous media with log-normal function fits to the observed DLS patterns to estimate d_avg_. (**c**) The zeta potential curves of PEI1200- and PEI60000-coated ultrasmall Ho_2_O_3_ nanoparticles in aqueous media. (**d**) Tyndall effect (or light scattering by the nanoparticle colloids) of PEI1200- and PEI60000-coated ultrasmall Ho_2_O_3_ nanoparticles in aqueous media (samples on the middle and right-side, respectively), establishing the colloidal dispersion of PEI-coated nanoparticles in aqueous media; no such light scattering is observed in triple-distilled water (sample on the left). Arrows show laser light scattering by nanoparticle colloids.

Figure 3a shows the aqueous nanoparticle suspension samples of PEI1200- and PEI60000-coated ultrasmall Ho_2_O_3_ nanoparticles with a concentration of ~30 mM Ho. They exhibited well-dispersed colloidal suspensions of PEI-coated nanoparticles in aqueous media. The average hydrodynamic diameters (a_avg_) of the PEI1200- and PEI60000-coated ultrasmall Ho_2_O_3_ nanoparticles were estimated to be 30.1 and 52.5 nm, respectively, from the log-normal function fits to the observed DLS patterns (Figure 3b and Table 1). The a_avg_ values were larger than the d_avg_ values because of the hydrophilic PEI coating and the accompanying hydration by water molecules around the nanoparticles. The larger a_avg_ of the PEI60000-coated nanoparticles can be attributed to their considerably lower grafting density due to the larger size of PEI60000 than that of PEI1200 (~50 times larger in M_n_). As shown in the HRTEM image (Figure 2b), each PEI60000 appeared to coat approximately two nanoparticles, which is quantitatively discussed in the surface-coating analysis in Section 3. This supports the observed larger a_avg_ of the PEI60000-coated nanoparticles in DLS patterns. Furthermore, the broader DLS pattern of the PEI60000-coated nanoparticles is attributed to the higher polydispersity [48] (i.e., molecular weight broadness = M_w_/M_n_ = 12.5) of PEI60000 compared with that (M_w_/M_n_ = 1.1) of PEI1200. The positive zeta potentials (ζ) of 19.9 and 20.7 mV of the PEI1200- and PEI60000-coated ultrasmall Ho_2_O_3_ nanoparticles (Figure 3c and Table 1), respectively, are due to the amine groups of PEI [49]. The high ζ values indicated the good colloidal stability of PEI-coated nanoparticles in aqueous media. The Tyndall effect (or light scattering by the nanoparticle colloids) was observed only for PEI1200- and PEI60000-coated nanoparticles (samples on the middle and right-side in Figure 3d, respectively) but not for triple-distilled water (sample on the left), establishing the colloidal dispersion of PEI-coated nanoparticles in aqueous media. 

**Figure 4:**
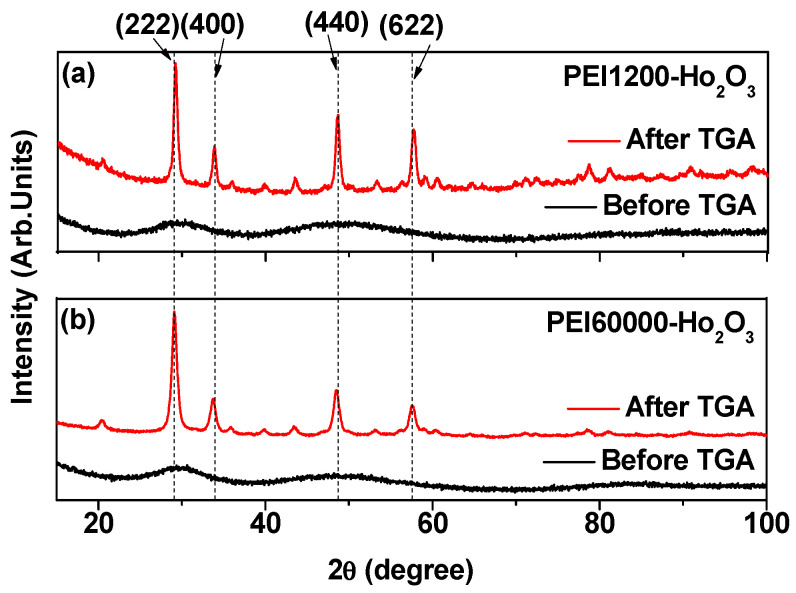
XRD patterns before (i.e., as-prepared) and after TGA of (**a**) PEI1200- and (**b**) PEI60000-coated ultrasmall Ho_2_O_3_ nanoparticles. The (222), (400), (440), and (622) assignments on the XRD peaks after TGA are the (hkl) Miller indices of cubic Ho_2_O_3_. All peaks after TGA are assigned with the (hkl) Miller indices of cubic Ho_2_O_3_.

The crystal structures of the as-prepared nanoparticles before and after TGA were determined via XRD analysis (Figure 4). Both samples demonstrated broad and amorphous XRD patterns before TGA, possibly because of their ultrasmall size [47]. However, the XRD patterns after TGA exhibited sharp peaks of body-centered cubic (bcc) Ho_2_O_3_. This was attributed to both particle size and crystal growth during TGA up to 900 °C. The lattice constant of TGA-treated powder samples was estimated to be 10.607 Å, which agreed with the reported value of 10.606 Å (Card No. 01-074-1829) [50]. 

### 3.2. Surface-Coating Results

**Figure 5:**
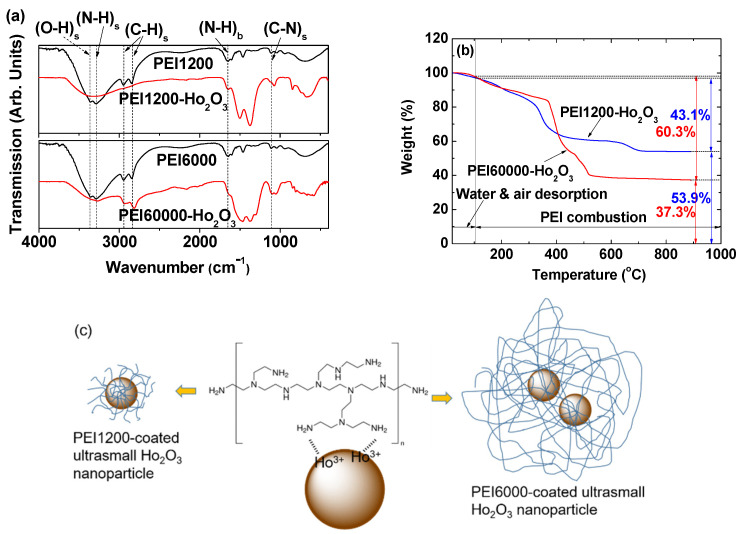
(**a**) FT-IR absorption spectra of PEI1200 and PEI60000 and PEI1200- and PEI60000-coated ultrasmall Ho_2_O_3_ nanoparticles. Subscripts “s” and “b” indicate stretching and bending vibrations, respectively. (**b**) TGA curves of PEI1200- and PEI60000-coated ultrasmall Ho_2_O_3_ nanoparticles. (**c**) PEI-coating structure: each nanoparticle is grafted with approximately fifteen PEI1200 polymers (left), multiple hard acid–hard base type of bondings (middle), and approximately two nanoparticles grafted with one PEI60000 polymer (left).

The PEI surface coating of the nanoparticles was examined using FT-IR absorption spectroscopy. As shown in Figure 5a, the characteristic IR absorption bands of PEI [35,49], such as the N-H stretching at 3292–3248 cm^−1^, C–H stretching at 2910–2950 cm^−1^, N-H bending at 1620–1660 cm^−1^, and C-N stretching at 1100–1200 cm^−1^, were observed in the FT-IR absorption spectra of PEI-coated nanoparticles, indicating the presence of PEI on nanoparticle surfaces. The N-H stretching and bending peaks overlap with the water stretching and bending peaks, respectively. Table 2 provides the details of the observed FT-IR absorption frequencies.

The amount of coating (P, wt.%) of PEI on the nanoparticle surfaces was determined based on the mass loss seen in the TGA curve (Figure 5b) after considering the initial mass loss because of water and air desorption between room temperature and ~105 °C. The estimated *p* values were 43.1 and 60.3% for the PEI1200- and PEI60000-coated nanoparticles, respectively (Table 1). The wt.% of Ho_2_O_3_ nanoparticles in the sample was estimated from the residual mass seen in the TGA curve. The grafting density (σ) [51], corresponding to the average number of PEI polymers coating a unit surface area of a nanoparticle, was estimated to be 1.15 and 0.0432 nm^−2^ for PEI1200- and PEI60000-coated nanoparticles, respectively, using the bulk density of Ho_2_O_3_ (8.41 g/cm^3^) [52], d_avg_ of the nanoparticles estimated by HRTEM, and the *p* value estimated from the TGA curve. The average number (N_NP_) of PEI polymers coating a nanoparticle was estimated by multiplying σ by the nanoparticle surface area (=πd^2^_avg_). From Table 1, it can be seen that σ and N_NP_ values decrease as the ligand’s size increases from PEI1200 to PEI60000 because the larger PEI60000 occupies a larger surface area owing to its higher steric effects than PEI1200. The surface-coating results are presented in Table 1.

PEI is grafted on the nanoparticle surface via a hard acid (Ho^3+^ on the nanoparticle surface)–hard base (NH_2_ of PEI) type of bonding [53]. Because each PEI has many NH_2_ groups, multiple bondings to each nanoparticle are possible (middle in Figure 5c). The estimated N_NP_ of ~15 and ~0.49 for PEI1200- and PEI60000-coated nanoparticles, respectively, indicate that each nanoparticle was grafted with approximately fifteen PEI1200 polymers (left in Figure 5c), whereas ~2 nanoparticles were grafted with a single PEI60000 polymer (right in Figure 5c). These results were substantiated by HRTEM images, demonstrating that individual nanoparticles were mostly observed for PEI1200-coated ultrasmall Ho_2_O_3_ nanoparticles (Figure 2a), whereas multiple paired nanoparticles were observed for PEI60000-coated ultrasmall Ho_2_O_3_ nanoparticles (Figure 2b).

### 3.3. In Vitro Cellular Cytotoxicity Results

**Figure 6:**
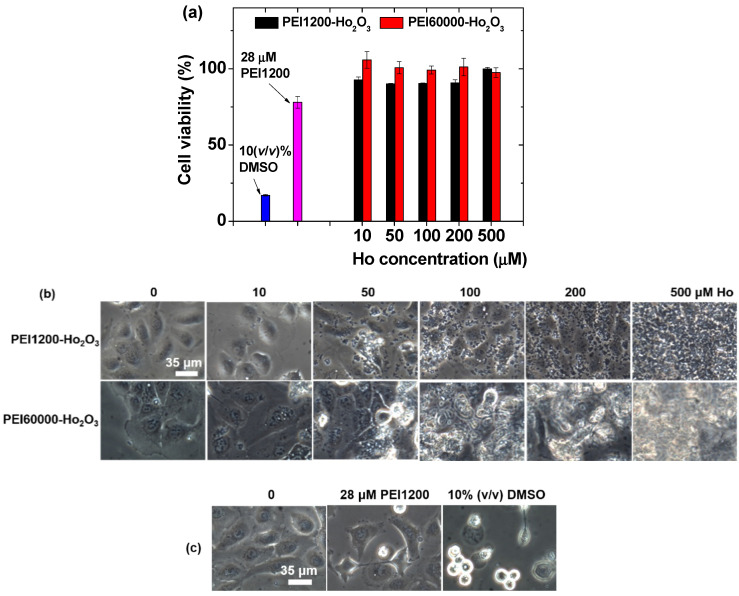
(**a**) In vitro cell viabilities after normalization with untreated control cells (0.0 mM Ho). 10% (*v*/*v*) DMSO was used as a positive control. (**b**) Optical microscopy images of the DU145 cells 48 h after incubation with PEI1200- and PEI60000-coated ultrasmall Ho_2_O_3_ nanoparticles. (**c**) Optical microscopy images of the DU145 cells 48 h after incubation with 10% (*v*/*v*) DMSO and 28 μM PEI1200.

The biocompatibility of PEI1200- and PEI60000-coated ultrasmall Ho_2_O_3_ nanoparticles was examined by measuring in the vitro cell viability of DU145 cells 48 h after incubation with the nanoparticle samples (Figure 6a). The results demonstrated very low cellular toxicities of up to 500 μM Ho for both the samples. Moreover, optical microscopy images of DU145 cells indicated that the PEI-coated nanoparticles were scattered across the cells, and many of them gathered around the cells (Figure 6b) owing to the electrostatic interaction between the positively charged amine groups of the PEI-coated nanoparticles (see the positive zeta potentials of PEI-coated nanoparticles in Table 1) and negatively charged cell membranes [54]. The nanoparticle coverage over the cells increased with an increase in nanoparticle concentration. However, the cell morphologies did not change with increasing nanoparticle concentration, possibly because of the very low cytotoxicity of PEI-coated nanoparticles. Moreover, 10% (*v*/*v*) DMSO diluted in the RPMI1640 cell culture medium exhibited cellular toxicity, thus serving as a positive control (Figure 6a). To determine any effect of free PEI polymers on cellular cytotoxicity, we measured the cell viability of PEI1200 at 28 μM PEI concentration, which corresponds to the PEI1200 concentration at 500 μM Ho concentration in the sample. As shown in Figure 6a, 28 μM PEI1200 was slightly toxic with a cell viability of 78% possibly due to membrane damage or DNA damage after the internalization of the polymers into the cells as investigated by others [55,56]. This indicates that free PEI polymers should be thoroughly washed out from the samples after surface coating. The observed very low cellular toxicities of the PEI1200- and PEI60000-coated nanoparticles are possibly due to their reduced interaction with the cells because of bindings of multiple −NH_2_ groups in each PEI1200 to a nanoparticle, resulting in reduced membrane damage and reduced internalization into the cells compared with free PEI polymers. The PEI1200 concentration was estimated as follows: PEI1200-coated Ho_2_O_3_ nanoparticles (d_avg_ = 2.05 nm; Table 1) contain ~269 Ho^3+^ ions [11], and the PEI concentration was roughly estimated as (500/269) × N_NP_ in which N_NP_ = 15 (Table 1). Optical microscopy images of DU145 cells after incubation with 10% (*v*/*v*) DMSO and 28 μM PEI1200 clearly exhibited a considerably reduced cell density for 10% *v*/*v*) DMSO as a positive control and a slight cell density reduction for 28 μM PEI1200 (Figure 6c). 

### 3.4. Magnetic Properties

**Figure 7:**
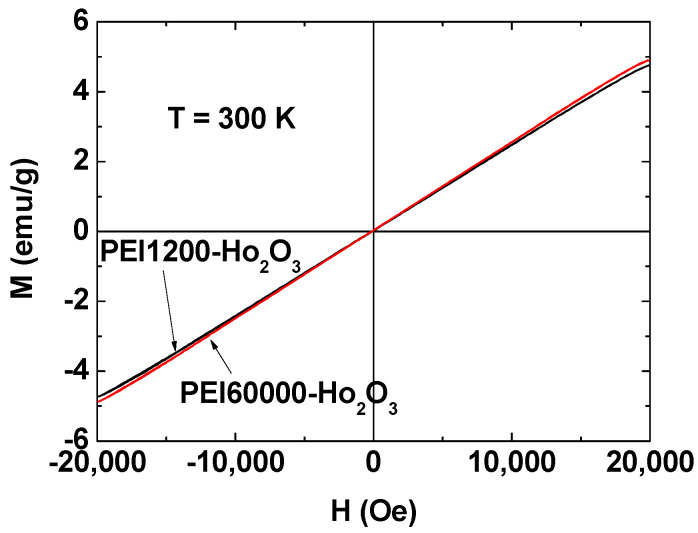
Mass-corrected M–H curves of the PEI1200- and PEI60000-coated ultrasmall Ho_2_O_3_ nanoparticles at 300 K obtained using the net masses of Ho_2_O_3_ nanoparticles without PEI, which were estimated from the TGA curves.

The magnetic properties of PEI-coated ultrasmall Ho_2_O_3_ nanoparticles were determined by measuring M–H curves (−2.0 T ≤ H ≤ 2.0 T) at 300 K using a vibrating sample magnetometer (Figure 7). Both the nanoparticle samples were paramagnetic and exhibited no hysteresis, zero coercivity, and zero remanence in the M–H curves, which is similar to bulk Ho_2_O_3_ [57,58]. The measured M values were mass-corrected using the net masses of the Ho_2_O_3_ nanoparticles without PEI, which were obtained from the net masses of the Ho_2_O_3_ nanoparticles in the TGA curves. From the mass-corrected M–H curves (Figure 7), the unsaturated net M values of the Ho_2_O_3_ nanoparticles without PEI at H = 2.0 T were estimated as 4.75 and 4.90 emu/g for PEI1200- and PEI60000-coated ultrasmall Ho_2_O_3_ nanoparticles, respectively (Table 3). Therefore, the average net M value of ultrasmall Ho_2_O_3_ nanoparticles without PEI was 4.83 emu/g.

### 3.5. r_1_ and r_2_ Values and R_1_ and R_2_ Map Images

**Figure 8:**
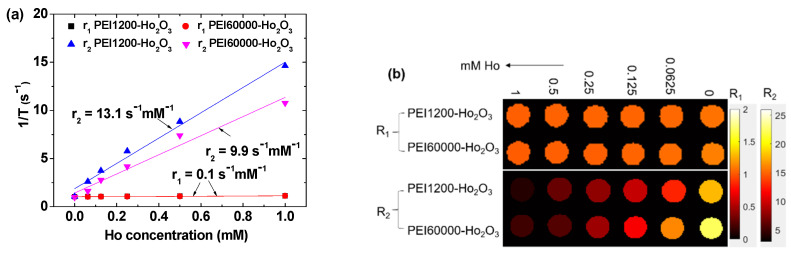
(**a**) Plots of inverse relaxation times 1/T_1_ and 1/T_2_ as a function of Ho concentration for PEI1200- and PEI60000-coated ultrasmall Ho_2_O_3_ nanoparticles in aqueous media at 3.0 T and 22 °C; the slopes yield r_1_ and r_2_ values, respectively. (**b**) R_1_ and R_2_ map images.

r_1_ and r_2_ water proton spin relaxivities and R_1_ and R_2_ map images were measured in an MR field of H = 3.0 T. r_1_ and r_2_ values were estimated from the slopes of the plots of inverse relaxation times 1/T_1_ and 1/T_2_ as a function of Ho concentration, respectively (Figure 8a). Both samples demonstrated negligible r_1_ (i.e., 0.1 s^−1^mM^−1^) and appreciable r_2_ values (13.1 and 9.9 s^−1^mM^−1^ for PEI1200- and PEI60000-coated ultrasmall Ho_2_O_3_ nanoparticles, respectively; Table 3). Consequently, the dose-dependent contrast enhancements observed in the R_1_ and R_2_ map images were negligible and appreciable, respectively, for both samples (Figure 8b). These results demonstrate in vitro that both samples can exclusively induce only T_2_ water proton spin relaxations and, thus, efficiently provide negative contrasts in MR images [2].

## 4. Discussion

In this study, PEI1200- and PEI60000-coated ultrasmall Ho_2_O_3_ nanoparticles (d_avg_ = 2.05 and 1.90 nm, respectively) were synthesized using a one-pot polyol method. In this method, the synthesis of ultrasmall Ho_2_O_3_ nanoparticles and surface coating with PEI were achieved via a one-step process in one pot. This method is simple and very useful for preparing hydrophilic and biocompatible ligand-coated ultrasmall lanthanide oxide nanoparticles with biomedical applications. PEI1200 and PEI60000 (M_n_ = 1200 and 60,000 amu, respectively) were used in this study to investigate the ligand-size effects on physicochemical properties and r_2_ values. The physicochemical properties of the synthesized PEI1200- and PEI60000-coated nanoparticles were characterized using various experimental techniques. Positive zeta potentials (~20 mV) were observed due to PEI coating for both the nanoparticle samples. The grafting density analyses [51] suggested that each nanoparticle was grafted with approximately fifteen PEI1200 polymers, whereas ~2 nanoparticles were grafted with a single PEI60000 polymer due to a considerably large molecular size of PEI60000; this finding was supported by HRTEM images (Figure 2a,b). Consequently, a larger hydrodynamic diameter (i.e., 52.5 nm) of PEI6000-coated nanoparticles than that (i.e., 30.1 nm) of the PEI1200-coated nanoparticles was observed. Moreover, a broader hydrodynamic diameter distribution in the DLS pattern of PEI6000-coated nanoparticles was observed than that of PEI1200-coated nanoparticles because of a higher polydispersity (i.e., 12.5) of PEI60000 than that (i.e., 1.1) of PEI1200. Both nanoparticle samples demonstrated good colloidal stability in aqueous media and promoted good DU145 cell viability. Interestingly, the development of T_2_ MRI contrast agents composed of ultrasmall nanoparticles that are excretable via the renal system, similarly to the case of molecular agents, is of considerable interest because the conventional iron oxide nanoparticles do not afford this possibility due to their appreciable particle sizes. For renal excretion, the nanoparticle diameter should be less than 3 nm [17,18].

Both nanoparticle samples show negligible r_1_ and appreciable r_2_ values (Table 3), which can be explained as follows. Negligible r_1_ values (i.e., 0.1 s^−1^mM^−1^) or negligible T_1_ water proton spin relaxation inductions can be explained by the inefficient interactions between the fast 4f-electron orbital motions of Ho^3+^ and slow water proton spin motions [19] as per the inner sphere model [4]. However, appreciable r_2_ values or appreciable T_2_ water proton spin relaxation inductions, which are caused by the fluctuation of local magnetic fields generated by the nanoparticle magnetic moments as per the outer sphere model [3,4], can be explained by the observed appreciable nanoparticle magnetic moment at room temperature (i.e., 4.83 emu/g at 2.0 T; Table 3).

The r_2_ value depends on multiple factors such as the solvent, sample solution pH, applied MR field, particle diameter, temperature, and surface-coating ligand [3,4,7,8]. All the factors except ligand size and particle diameter were similar for these two nanoparticle samples. The PEI60000-coated nanoparticles have a thicker coating than PEI1200-coated nanoparticles because each nanoparticle is grafted with an average PEI-mass of 18,000 and 29,400 amu in the case of PEI1200- and PEI60000-coated nanoparticles, respectively, as seen from their N_NP_ of ~15 and ~0.49 (Table 1), respectively. Note that r_2_ is proportional to M_NP_^2^/L^3^, where M_NP_ is the magnetic moment per nanoparticle (unit: emu/nanoparticle) and L is the distance between the nanoparticles and water proton spins [3,4]. Here, M_NP_ = ~μ(Ho^3+^) (d_avg_/0.234)^3^, where μ(Ho^3+^) is the atomic magnetic moment of Ho^3+^ [11], and L is proportional to ligand-coating thickness. Therefore, using d_avg_ values (Table 1), the M_NP_ of the PEI1200-coated nanoparticles is ~1.26 times that of the PEI60000-coated nanoparticles and the L value of the former nanoparticles is shorter than that of the latter nanoparticles. This roughly explains why the observed r_2_ value of the PEI1200-coated nanoparticles is higher than that of the PEI60000-coated nanoparticles.

Note that T_1_ water proton spin relaxation always accompanies T_2_ water proton spin relaxation, whereas the reverse is not true [59]. Therefore, for the observed negligible r_1_ and appreciable r_2_ values or very high r_2_/r_1_ ratios, the PEI1200- and PEI60000-coated ultrasmall Ho_2_O_3_ nanoparticles can generate negative contrasts in MR images even with only appreciable (i.e., not high) r_2_ values because their T_1_ water proton spin relaxation contribution to MR images is negligible [2]. Thus, they are efficient T_2_ MRI contrast agents. This hypothesis was confirmed in vitro from the negligible and appreciable dose-dependent contrast enhancements in R_1_ and R_2_ map images, respectively (Figure 8b). 

## 5. Conclusions

PEI1200- and PEI60000-coated ultrasmall Ho_2_O_3_ nanoparticles (d_avg_ = 2.05 and 1.90 nm, respectively) were synthesized using a one-pot polyol method and characterized using multiple experimental techniques. Their r_1_ and r_2_ values and R_1_ and R_2_ map images were measured to explore their potential as efficient T_2_ MRI contrast agents in vitro.

(1) Both nanoparticle samples demonstrated low cellular cytotoxicity and good colloidal stability owing to the PEI coating on the nanoparticle surfaces.
(2) Appreciable r_2_ values of 13.1 s^−1^mM^−1^ for the PEI1200-coated ultrasmall Ho_2_O_3_ nanoparticles and 9.9 s^−1^mM^−1^ for the PEI60000-coated ultrasmall Ho_2_O_3_ nanoparticles were observed. Negligible r_1_ values of 0.1 s^−1^mM^−1^ were observed for both nanoparticle samples. Consequently, R_1_ map images with negligible dose-dependent contrast changes and R_2_ map images with appreciable dose-dependent contrast changes were obtained for both nanoparticle samples. These in vitro experimental results demonstrate that PEI1200- and PEI60000-coated ultrasmall Ho_2_O_3_ nanoparticles can act as efficient T_2_ MRI contrast agents. In vivo MRI studies will further demonstrate the potential of ultrasmall Ho_2_O_3_ nanoparticles as efficient T_2_ MRI contrast agents.

## Figures and Tables

**Table 1:** Summary of the observed physicochemical properties of PEI1200- and PEI60000-coated ultrasmall Ho_2_O_3_ nanoparticles.

Ligand	d_avg_ (nm)	a_avg_ (nm)	Sample Solution pH	ζ (mV)	Surface-Coating Result		
					P ^a^ (wt.%)	σ ^b^ (L/nm^2^)	N_NP_^c^
PEI1200	2.05	30.1	7.0−7.5	19.9	43.1	1.15	15
PEI60000	1.90	52.5	7.0–7.5	20.7	60.3	0.0432	0.49

^a^ Average surface-coating amount in wt.% per nanoparticle. ^b^ Average grafting density (i.e., average number of PEI polymers coating a nanoparticle unit surface area). ^c^ Average number of PEI polymers coating a nanoparticle.

**Table 2:** Observed FT-IR absorption frequencies (in cm^−1^) ^a^.

	(O-H) _s_	(N-H) _s_	(C-H) _s_	(N-H) _b_	(C-N) _s_
PEI1200	3360	3291	29,452,833	1654	1115
PEI60000	3362	3291	29,452,833	1656	1106
PEI1200-Ho_2_O_3_	-	-	2970	1649	1112
PEI60000-Ho_2_O_3_	-	3269	29,462,822	1650	1110

^a^ Subscripts “s” and “b” indicate stretching and bending vibrations, respectively.

**Table 3:** Magnetic properties and water proton spin relaxivities.

Nanoparticle	Magnetic Properties at 300 K		Water Proton Spin Relaxivities (s^−1^mM^−1^) at 22 °C and 3.0 T	
	Magnetism	Net M (emu/g) at 2 T	r_1_	r_2_
PEI1200-Ho_2_O_3_	Paramagnetism	4.75	0.1	13.1
PEI60000-Ho_2_O_3_	Paramagnetism	4.90	0.1	9.9

## Data Availability

The data presented in this study are available upon request from the corresponding authors.
